# Palmitoylation and membrane cholesterol stabilize μ-opioid receptor homodimerization and G protein coupling

**DOI:** 10.1186/1471-2121-13-6

**Published:** 2012-03-19

**Authors:** Hui Zheng, Elizabeth A Pearsall, Dow P Hurst, Yuhan Zhang, Ji Chu, Yali Zhou, Patricia H Reggio, Horace H Loh, Ping-Yee Law

**Affiliations:** 1 Department of Pharmacology, University of Minnesota, Minneapolis, Minnesota 55455; 2 Stem Cell and Cancer Biology Group, Key Laboratory of Regenerative Biology, South China Institute for Stem Cell Biology and Regenerative Medicine, Guangzhou Institutes of Biomedicine and Health, Chinese Academy of Sciences, Guangzhou 510530, China; 3 Department of Chemistry and Biochemistry, Center for Drug Discovery, University of North Carolina, Greensboro, North Carolina 27402

**Keywords:** Palmitoylation, Cholesterol, Homodimerization, G protein coupling

## Abstract

**Background:**

A cholesterol-palmitoyl interaction has been reported to occur in the dimeric interface of the β_2_-adrenergic receptor crystal structure. We sought to investigate whether a similar phenomenon could be observed with μ-opioid receptor (OPRM1), and if so, to assess the role of cholesterol in this class of G protein-coupled receptor (GPCR) signaling.

**Results:**

C3.55(170) was determined to be the palmitoylation site of OPRM1. Mutation of this Cys to Ala did not affect the binding of agonists, but attenuated receptor signaling and decreased cholesterol associated with the receptor signaling complex. In addition, both attenuation of receptor palmitoylation (by mutation of C3.55[170] to Ala) and inhibition of cholesterol synthesis (by treating the cells with simvastatin, a HMG-CoA reductase inhibitor) impaired receptor signaling, possibly by decreasing receptor homodimerization and Gαi2 coupling; this was demonstrated by co-immunoprecipitation, immunofluorescence colocalization and fluorescence resonance energy transfer (FRET) analyses. A computational model of the OPRM1 homodimer structure indicated that a specific cholesterol-palmitoyl interaction can facilitate OPRM1 homodimerization at the TMH4-TMH4 interface.

**Conclusions:**

We demonstrate that C3.55(170) is the palmitoylation site of OPRM1 and identify a cholesterol-palmitoyl interaction in the OPRM1 complex. Our findings suggest that this interaction contributes to OPRM1 signaling by facilitating receptor homodimerization and G protein coupling. This conclusion is supported by computational modeling of the OPRM1 homodimer.

## Background

A cholesterol-palmitoyl interaction at C7.68(341) has been observed in the crystallographic dimeric interface of transmembrane helix (TMH) 1 and Helix 8 in the β_2_-adrenergic receptor (β_2_-AR) crystal structure [1]. Palmitoylation is a covalent attachment of palmitic acid to cysteine residues of membrane proteins. Palmitoylation of the rhodopsin sub-family of G protein-coupled receptors (GPCRs) has been universally reported, and similar cholesterol-palmitoyl interactions may exist within other GPCRs. Sequence alignment has identified cysteine residues in the carboxy termini as potential palmitoylation sites in about 78% of 74 GPCRs examined [2]. However, these cysteines are not the only palmitoylation sites. For example, although rat μ-opioid receptor (OPRM1) has two cysteines [C7.63(346) and C7.68(351)] in its C terminus, mutation of these cysteines did not decrease the palmitoylation of OPRM1 [3], suggesting that C3.55(170) (the only other intracellular cysteine of rat OPRM1) may be the palmitoylation site. Similarly, V_1A _vasopressin receptor also has palmitoylation sites outside its C terminal domain [4]. Normally, palmitoylation facilitates the membrane targeting and signaling of GPCRs [5]. For instance, palmitoylation-dependent receptor-G protein interaction is observed with both the β_2_-adrenergic receptor and the M2 muscarinic acetylcholine receptor [6,7].

Although there is no definitive answer as to how receptor palmitoylation contributes to GPCR signaling, the cholesterol-palmitoyl interaction at the β_2_-AR crystallographic dimeric interface suggests that facilitation of homodimerization may be one possible scenario. Because of the enrichment of many GPCRs in lipid raft (cholesterol-rich) microdomains in cell membranes [8], cholesterol within such microdomains can be easily incorporated into the receptor dimer. In addition, because the interaction surface appears to be too small for the GPCR monomer to interact with G proteins [9], dimerization may facilitate G protein coupling. In fact, dimerization of many GPCRs, including OPRM1 and β_2_-AR, regulates receptor signaling [10].

In the work described here, we tested the hypothesis that a specific cholesterol-palmitoyl interaction within the OPRM1 signaling complex affects its signaling by facilitating homodimerization and G protein coupling. Cholesterol, an important component of lipid raft microdomains on the cell membrane, is critical for GPCR signaling [11], and the localization of some GPCRs in lipid raft microdomains has been suggested to contribute to downstream signaling [8]. For example, OPRM1 locates in lipid raft microdomains on the cell membrane in the absence of agonist [12]. Extraction of cholesterol from cells disrupts the entirety of the lipid raft microdomains and inhibits OPRM1 signal transduction in both morphine-induced adenylyl cyclase inhibition and ERK phosphorylation [12]. Thus, if a cholesterol-palmitoyl interaction could be identified in the interface of the OPRM1 homodimer, it would suggest that cholesterol and cholesterol-enriched lipid raft microdomains may be linked to receptor palmitoylation during regulation of receptor signaling. Further, if the involvement of receptor dimerization and G protein coupling could be determined, this finding would extend our understanding of the mechanisms that underlie GPCR signaling.

We identified the palmitoylation site on OPRM1 and examined the ability of the cholesterol-palmitoyl interaction to contribute to receptor homodimerization, G protein coupling, and signaling. In addition, we developed a computational model of OPRM1 to calculate the contribution of the cholesterol-palmitoyl interaction to the total interaction energy at the homodimer interface.

## Results

### Cys^170 ^is the palmitoylation site of OPRM1

We used wild-type HEK293 cells (HEK) and HEKOPRM1 cells (HEK cells heterologously expressing OPRM1 with HA spliced at the amino terminus) to validate the palmitoylation assay [13]. HA-tagged receptors were precipitated with HA antibody and protein G agarose. The following procedures were used to determine receptor palmitoylation: 1) Free sulfhydryl groups in precipitated receptors were blocked with N-ethylmaleimide (NEM). 2) Palmitoylated cysteines were hydrolyzed with hydroxylamine. 3) Biotin was conjugated to the de-palmitoylated cysteines in the immunoprecipitated receptors with btn-BMCC (1-biotinamido-4- [4'- (maleimidomethyl) cyclohexane carboxamido] butane). The amount of biotin linked to the receptors was determined by immunoblotting.

**Figure 1:**
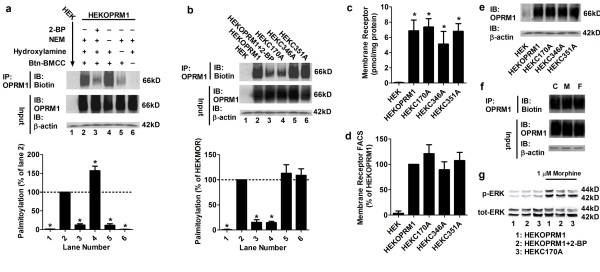
**Cys^170 ^is the palmitoylation site of OPRM1**. (a) Palmitoylation assays were performed in HEK and HEKOPRM1 cells. The amounts of palmitoylated receptor were normalized against that in HEKOPRM1 (Lane 2). 50 μM 2-BP was used to treat HEKOPRM1 for 12 h (Lane 3). Individual steps (treatment with NEM, hydroxylamine, or btn-BMCC) were omitted to validate the assay (Lanes 4-6). (b) The palmitoylation assay was performed in HEK, HEKOPRM1, HEKOPRM1 treated with 50 μM 2-BP for 12 h, HEKC170A, HEKC346A, and HEKC351A cells. The amounts of palmitoylated receptor were normalized against that in HEKOPRM1 (Lane 2). (c-e) Membrane receptor levels were determined with binding assay (c), FACS (d), and immunoblotting (e). (f) HEKOPRM1 cells were treated with PBS (C), 1 μM morphine (M) and 10 nM fentanyl (F) for 5 min. Receptor palmitoylation was then determined. (g) HEKOPRM1, HEKOPRM1 treated with 50 μM 2-BP for 12 h, and HEKC170A cells were treated with 1 μM morphine for 5 min. Phosphorylated ERK and total-ERK were determined by immunoblotting. One-way ANOVA with Dunnett's test as a post-hoc test was used for analysis. The error bars and "*" represent the standard deviations and significant changes (p < 0.05, n > 3), respectively.

We observed intensive biotin labeling in the HEKOPRM1 cells but not in the HEK cells, which suggested that the palmitoylation we detected was specific to OPRM1 (Figure 1a, Lanes 1-2). Since NEM was used to block free sulfhydryl groups, the immunoreactivity of biotin increased when the NEM step was omitted (Figure 1a, Lane 4). In addition, the immunoreactivity of biotin decreased when the hydroxylamine step was omitted (Figure 1a, Lanes 5) and no biotin was detected when btn-BMCC was not used (Figure 1a, Lanes 6), which confirmed that the assay was suitable for detecting the palmitoylation of OPRM1. To further confirm that the assay could be used to detect palmitoylation specifically, we used 2-bromopalmitate (2-BP), a palmitoylation inhibitor, to block all palmitoylation. A low level of receptor palmitoylation was observed when HEKOPRM1 cells were pretreated with 2-BP for 12 h (Figure 1a, Lane 3), which also indicated that the palmitoylation we detected was on OPRM1. Since palmitoylation is important for cell function, prolonged treatment with or high concentrations of 2-BP will affect cell viability. In the current study, the treatment time and concentration of 2-BP were determined empirically so as to inhibit receptor palmitoylation while having minimal effect on cell viability. Thus, the 2-BP treatment paradigm we used did not completely block receptor palmitoylation.

Mutation of the two conserved cysteine residues (C8.53[346] and C8.58[351]) in the C terminus of OPRM1 does not affect palmitoylation [3]. Thus, we predicted the only other intracellular cysteine, C3.55(170), to be the putative palmitoylation site. To confirm this hypothesis, each of these three cysteines was mutated to alanine individually, and the mutants were stably expressed in HEK cells to obtain the following: HEKC170A, HEKC346A, and HEKC351A. Although C170A is difficult to stably express in CHO cells [3], we were able to successfully express a high level of C170A in the cell membrane of HEK cells, possibly because of differences between cell lines or our use of poly-L-lysine during cell culture. As shown in Figure 1b, Lanes 2 and 5-6, we detected similar amounts of palmitoylated receptor in HEKOPRM1, HEKC346A, and HEKC351A cells. Furthermore, the amount of palmitoylated receptor in HEKC170A cells was similar to that in 2-BP-pretreated HEKOPRM1 cells (Figure 1b). These results suggest that C3.55(170) but not C8.53(346) or C8.58(351) is indeed the palmitoylation site of OPRM1.

Our subsequent [^3^H]-diprenorphine saturation binding assay using isolated cell membranes indicated that there was no difference in the amounts of receptors in the cell membranes of the HEKOPRM1, HEKC170A, HEKC346A, and HEKC351A cells (membrane receptors were expressed in HEK, HEKOPRM1, HEKC170A, HEKC346A, and HEKC351A cells at 0.06 ± 0.05, 6.87 ± 1.14, 7.36 ± 1.10, 5.12 ± 1.67, and 6.80 ± 1.61 pmol/mg protein respectively [Figure 1c]). Our FACS analysis using an antibody against the HA-tag further confirmed that there was no difference in the amounts of membrane receptor between the four cell lines (Figure 1d). Since the HA-tag was located in the receptor N-terminus, and the cell membrane was not disrupted during the analysis, the results obtained from the FACS assay should represent the actual amounts of membrane receptor. Lastly, immunoblotting also indicated that the overall receptor expression levels were similar between the four cell lines (Figure 1e). In light of these results, it is reasonable to conclude that C3.55(170) is the major palmitoylation site of OPRM1. For the sake of consistency, we will now use "OPRM1" to refer to the wild-type OPRM1, "C170A" to refer to the palmitoylation-deficient mutant, and "receptor" to indicate both the wild type and mutants.

Of note, agonist treatment did not affect receptor palmitoylation when morphine and fentanyl were used to challenge the HEKOPRM1 cells (Figure 1f). Since the studies described here focused on how receptor palmitoylation influences receptor signaling, the effects of agonists on receptor palmitoylation or other subsequent observations are not discussed in depth.

### Receptor palmitoylation stabilizes morphine-induced signaling and receptor-Gαi2 coupling

To determine the influence of palmitoylation on receptor signaling, we monitored morphine-induced adenylyl cyclase inhibition and ERK phosphorylation. Morphine-induced adenylyl cyclase inhibition is defined by the ability of morphine to inhibit the forskolin-induced increase in the intracellular cAMP level. We analyzed morphine-induced ERK phosphorylation by calculating the percentage increase of phosphorylated ERK when compared to basal level.

As summarized in Table 1, no difference in the affinities for ligands (morphine, naloxone, and Cys^2^-Tyr^3^-Orn^5^-Pen^7^-amide [CTOP]) was detected between OPRM1 and C170A. For example, the K_I _of CTOP was 11 ± 1.4 nM in HEKOPRM1 cells, and was 9.5 ± 2.1 nM in HEKC170A cells (Table 1). In addition, the expression levels of OPRM1 and C170A in the cell membrane were similar (Figure 1c-e). However, morphine induced less signaling in HEKC170A than in HEKOPRM1 cells. The ability of morphine to induce adenylyl cyclase inhibition in HEKC170A was approximately 75% of that in HEKOPRM1, when maximum inhibition was analyzed (Table 2). The ability to induce ERK phosphorylation in HEKC170A was approximately 69% of that in HEKOPRM1 (Table 2 and Figure 1g). Morphine also induced receptor signaling in 2-BP-treated HEKOPRM1 cells and in HEKC170A cells (Table 2 and Figure 1g). These results suggest that palmitoylation blockage impairs receptor signaling induced by morphine.

**Table 1:** Palmitoylation does not affect the binding of agonists.

		HEKOPRM1	HEKC170A
**Relative Affinities**			

**Morphine**	**K_H _(nM)**	2.8 ± 0.56	2.2 ± 0.42
	**K_L _(nM)**	155 ± 27	123 ± 31

**Naloxone**	**K_I _(nM)**	5.1 ± 0.71	6.2 ± 1.2

**CTOP**	**K_I _(nM)**	11 ± 1.4	9.5 ± 2.1

The affinities of receptor for ligands were determined in HEKOPRM1 and HEKC170A cells using an agonist binding assay. One-site (K_I_: naloxone and CTOP) or two-site (K_H _and K_L_: morphine) curve-fitting models were used in the analyses with GraphPad Prism 5.0. Data were analyzed by one-way ANOVA with post-hoc Dunnett's test. Standard deviations are provided, and "*" represents significant changes (p < 0.05).

**Table 2:** Palmitoylation impairs morphine-induced receptor signaling.

		HEKOPRM1	HEKOPRM1 +2-BP	HEKC170A
**Morphine-Induced Signaling**				

**AC Inhibition**	**K_I _(nM)**	9.3 ± 0.89	15 ± 1.2*	14 ± 1.3*
	**Max. Inh. (%)**	84 ± 2.8	56 ± 5.1*	63 ± 4.3*
**pERK**	**(% of basal)**	239 ± 10	154 ± 10*	164 ± 12*

The percentage inhibition of forskolin-induced cAMP increase by various concentrations of morphine was analyzed with GraphPad Prism 5.0. The results are presented as the maximum inhibition (Max. Inh.) and the IC50 values (K_I_). ERK phosphorylation was indicated by the amount of phosphorylated ERK after 5 min of treatment with 1 μM morphine (see blots provided in Figure 1g). The amount of total ERK and the results under control conditions were used for normalization. Experiments were repeated at least four times. Data were analyzed by one-way ANOVA with post-hoc Dunnett's test. Standard deviations are provided, and "*" represents significant changes (p < 0.05).

Both of the signaling events that we monitored are mediated via Gαi2 [12]. In addition, up-regulation and down-regulation of Gαi2 in HEKOPRM1 cells significantly affects adenylyl cyclase inhibition and ERK phosphorylation induced by morphine. Thus, we thought it likely that the impaired signaling described above was indicative of a decrease in receptor-Gαi2 coupling. We next investigated the effect of receptor palmitoylation on Gαi2 coupling.

**Figure 2:**
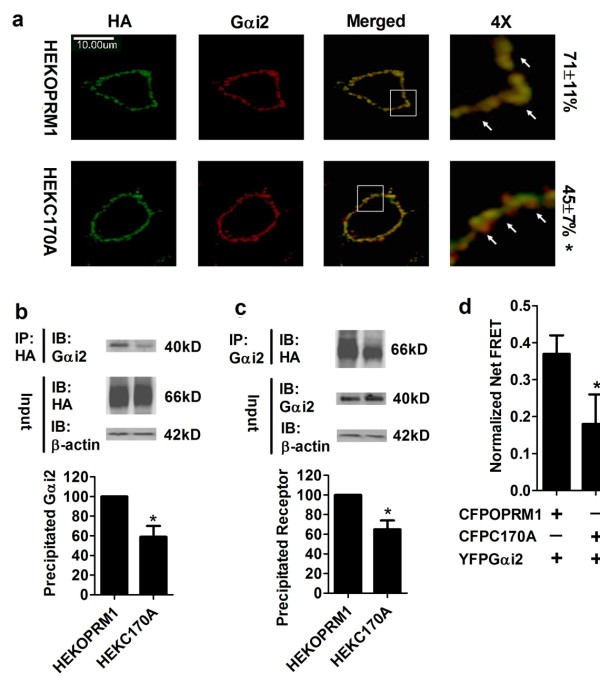
**Palmitoylation contributes to Gαi2 coupling**. (a) The colocalization between HA-tagged receptor and Gαi2 was determined in HEKOPRM1 and HEKC170A cells. Images were analyzed as described in *Methods*. (b) Anti-HA antibody was used to precipitate receptors in HEKOPRM1 and HEKC170A cells. Gαi2 precipitated with receptors was quantified and normalized to that in HEKOPRM1cells. (c) Co-immunoprecipitation was performed with Anti-Gαi2 antibody and the precipitated receptor was quantified. (d) CFPOPRM1 or CFPC170A was transfected into HEK cells with YFPGαi2. FRET analysis was performed. A two-tailed student t-test was used. Error bars and "*" represent the standard deviations and significant changes (p < 0.05, n > 3 for b and c; n > 10 for a and d), respectively. IP, immunoprecipitation; IB, immunoblotting.

When we analyzed the immunoreactivity of OPRM1 and Gαi2 on the cell membrane (Figure 2a), the colocalization between receptor and Gαi2 in HEKOPRM1 cells was more obvious than in HEKC170A cells. Similar observations were noted in our co-immunoprecipitation experiments (Figure 2b and 2c). When we used a Gαi2 antibody to perform co-immunoprecipitation, the amount of OPRM1 co-immunoprecipitated with Gαi2 was greater than that of C170A. When HA antibody was used to immunoprecipitate the receptor, more Gαi2 was co-immunoprecipitated with OPRM1 than with C170A. These results indicate that the interaction between Gαi2 and C170A is weaker than that between Gαi2 and OPRM1.

We further investigated the interaction between receptor and Gαi2 with FRET analysis. The normalized net FRET between CFPOPRM1 and YFPGαi2 was much higher than that between CFP and YFP, suggesting that OPRM1 and Gαi2 were in close proximity of each other (≤ 10 nm). We performed FRET analysis on the cell membrane to exclude any possible influence from the intracellular expression of fluorescence constructs. The normalized net FRET between CFPOPRM1 and YFPGαi2 was higher than that between CFPC170A and YFPGαi2 (Figure 2d). Because 1) we kept the expression of the fluorescence constructs, like CFPOPRMA1 and YFPGαi2 to similar levels by titrating the amounts of plasmids used for transfection, 2) we used immunoblotting to monitor expression during our studies, and 3) we determined overall fluorescence intensities prior to our FRET and colocalization studies, the FRET difference supports the conclusion that blockage of receptor palmitoylation in the C170A mutant impairs Gαi2 coupling.

The YFP/CFP tagged receptors had similar functions when either FLAG- or HA-tagged. Morphine-induced adenylyl cyclase inhibition in the cells caused transient expression of these receptor constructs with similar K_I_s: 9.8 ± 1.1 nM (HA-tagged OPRM1), 10.7 ± 1.4 nM (FLAG-tagged OPRM1), 8.9 ± 1.2 nM (CFPOPRM1), and 9.5 ± 0.8 nM (YFPOPRM1). Thus, the FRET experiments should be indicative of the functional characteristics of the receptors.

### Receptor palmitoylation facilitates homodimerization and subsequent Gαi2 coupling

**Figure 3:**
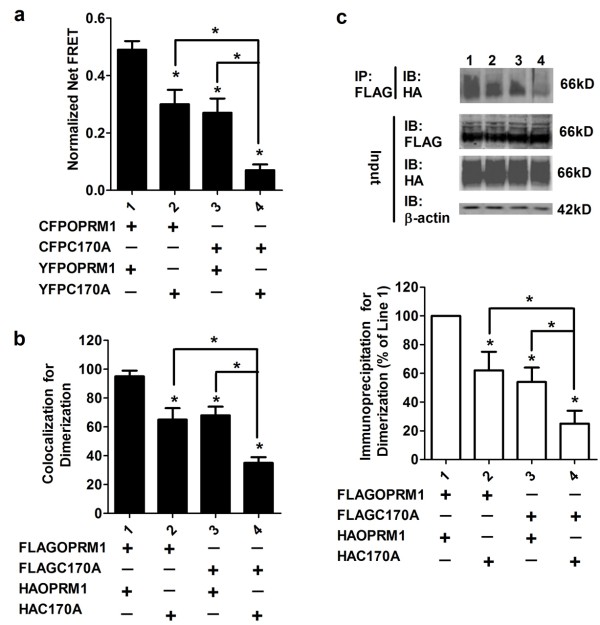
**Palmitoylation stabilizes homodimerization**. (a) FRET analysis was performed after transfecting combinations of CFPOPRM1/CFPC170A and YFPOPRM1/YFPC170A into HEK cells. (b-c) FLAGOPRM1/FLAGC170A and HAOPRM1/HAC170A were transfected into HEK cells. The colocalization between FLAG-tagged receptor and HA-tagged receptor was determined in (b). The amounts of HA-tagged receptor precipitated with Flag-tagged receptor were normalized against the amount of HAOPRM1 precipitated with FLAGOPRM1 and summarized in (c). One-way ANOVA with Dunnett's test as a post-hoc test was used for analysis. The error bars and "*" represent the standard deviations and significant changes (p < 0.05, n > 3), respectively.

We investigated the possible contribution of OPRM1 palmitoylation to homodimerization by performing FRET analysis between CFPOPRM1/CFPC170A and YFPOPRM1/YFPC170A. As indicated in Figure 3a, the normalized net FRET between CFPOPRM1 and YFPOPRM1 was 0.49 ± 0.03, whereas it was 0.07 ± 0.02 between CFPC170A and YFPC170A in the cell membrane. In addition, when the HEK cells were co-transfected with CFPOPRM1 and YFPC170A or with CFPC170A and YFPOPRM1, the normalized net FRETs were 0.30 ± 0.05 and 0.27 ± 0.05, respectively. The colocalization and co-immunoprecipitation assays between HAOPRM1/HAC170A and FLAGOPRM1/FLAGC170A confirmed the results of the FRET assay (Figure 3b and 3c). In summary, our colocalization, co-immunoprecipitation and FRET studies suggest that the amount of the OPRM1-OPRM1 homodimer is greater than the amount of the OPRM1-C170A dimer, and the amount of the OPRM1-C170A dimer is greater than that of the C170A-C170A homodimer, when similar levels of receptors are expressed. It is reasonable, therefore, to suggest that the ability of C170A to form a homodimer is lower than that of OPRM1.

**Figure 4:**
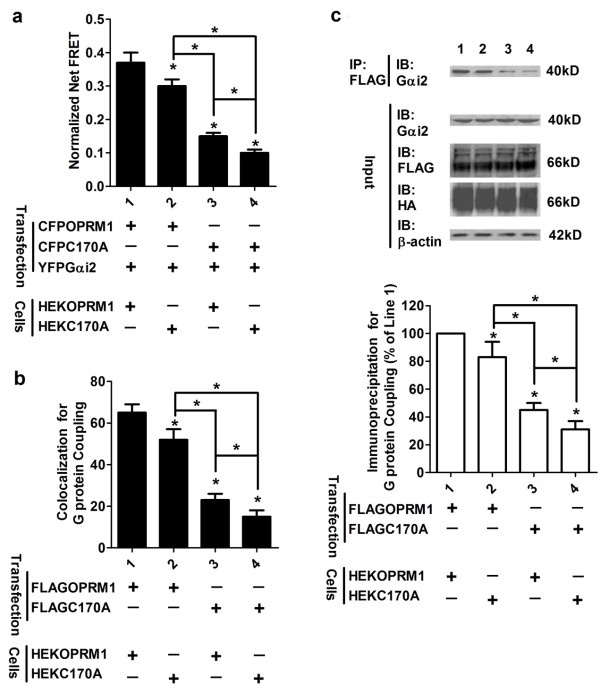
**Palmitoylation stabilizes Gαi2 coupling**. (a) The FRET between CFPOPRM1 and YFPGαi2 and the FRET between CFPC170A and YFPGαi2 were determined in HEKOPRM1 and HEKC170A cells. (b-c) Colocalization between FLAGOPRM1 and Gαi2 (or FLAGC170A and Gαi2) was compared in HEKOPRM1 and HEKC170A cells after transfection (b). The amounts of Gαi2 precipitated with FLAGOPRM1 and FLAGC170A in the two cell lines were compared in (c). The amount of Gαi2 precipitated with FLAGOPRM1 in HEKOPRM1 cells was used for normalization. One-way ANOVA with Dunnett's test as a post-hoc test was used. The error bars and "*" represent the standard deviations and significant changes (p < 0.05, n > 3), respectively.

Because the amounts of homodimer decreased sequentially from Lane 1 to Lane 4 in Figure 3c, we used FRET analysis to determine if the decrease affected receptor-Gαi2 coupling (Figure 4a). We transiently transfected YFPGαi2 with either CFPOPRM1 or CFPC170A into HEKOPRM1 and HEKC170A cells. We considered two caveats in these experiments and took the following steps to ensure the success of the studies: 1) we determined that HEKOPRM1 and HEKC170A cells expressed similar amounts of membrane receptors; and 2) we tightly controlled transient transfection of CFPOPRM1 and CFPC170A in order to reach similar expression levels.

According to our hypothesis, if receptor palmitoylation affects Gαi2 coupling, a similar sequential decrease in receptor-Gαi2 coupling should be observed between OPRM1 homodimer, OPRM1-C170A dimer and C170A homodimer. As indicated in Figure 4a, we observed that the normalized net FRET between CFPOPRM1 and YFPGαi2 was greater than that between CFPC170A and YFPGαi2 in both HEKOPRM1 and HEKC170A cells. The normalized net FRET between CFPOPRM1 and YFPGαi2, as well as between CFPC170A and YFPGαi2, was greater in HEKOPRM1 than in HEKC170A. These results suggest a positive correlation between the receptor palmitoylation and Gαi2 coupling.

This correlation can be explained by two potential mechanisms. One possibility is that the homodimer's affinity for Gαi2 is much higher than the monomer's affinity for Gαi2; this mechanism is supported by a previous report [9]. A second possibility is that the C170A monomer's affinity for Gαi2 is much lower than the OPRM1 monomer's affinity for Gαi2. If the second mechanism was the dominant one, the FRET between transiently transfected CFPC170A and YFPGαi2 would be smaller in HEKOPRM1 cells than in HEKC170A cells, because OPRM1's higher affinity for YFPGαi2 would result in a higher competition for Gαi2 in HEKOPRM1 than in HEKC170A. However, our FRET analysis produced the opposite result: the FRET between CFPC170A and YFPGαi2 was higher in HEKOPRM1 cells than in HEKC170A cells (Figure 4a). These observations suggest that the reduced receptor dimerization in the absence of palmitoylation leads to decreased Gαi2 coupling. Our additional colocalization and co-immunoprecipitation studies further supported this hypothesis (Figure 4b and 4c). In total, these results indicate a correlation between receptor homodimerization and Gαi2 coupling.

### Receptor palmitoylation facilitates cholesterol association in the receptor signaling complex

In order to determine the detailed mechanisms underlying these phenomena, we utilized the observed interaction between cholesterol and palmitoyl group in the crystal structure of β_2_-AR [1]. Before we could determine the existence of a similar cholesterol-palmitoyl interaction in the OPRM1 complex, however, we first needed to quantify the existing cholesterol in the receptor complex. Because direct detection of cholesterol within the homodimer requires purification of the receptor to homogeneity, and there is no guarantee that the cholesterol-receptor association will stay intact during purification, we instead examined the amount of cholesterol incorporated into the receptor signaling complex using a new method, described below.

To determine cholesterol association with the receptor complex, we used HA-antibody to precipitate the HA-tagged receptor. In this method, if cholesterol does associate with the receptor complex specifically, greater amounts of cholesterol should be precipitated by the HA antibody when compared to immunoprecipitation with no antibody. To avoid possible influence from the usage of antibody, FLAG antibody was used as control antibody, as no protein was FLAG-tagged in current paradigm. Cholesterol association with the receptor signaling complex was indicated by the additional amount of cholesterol precipitated by the HA antibody compared with that precipitated by a control antibody. Extensive washing with lysis buffer containing Triton X-100 and digitonin ensured the removal of cholesterol that was nonspecifically associated with the receptor signaling complex.

**Figure 5:**
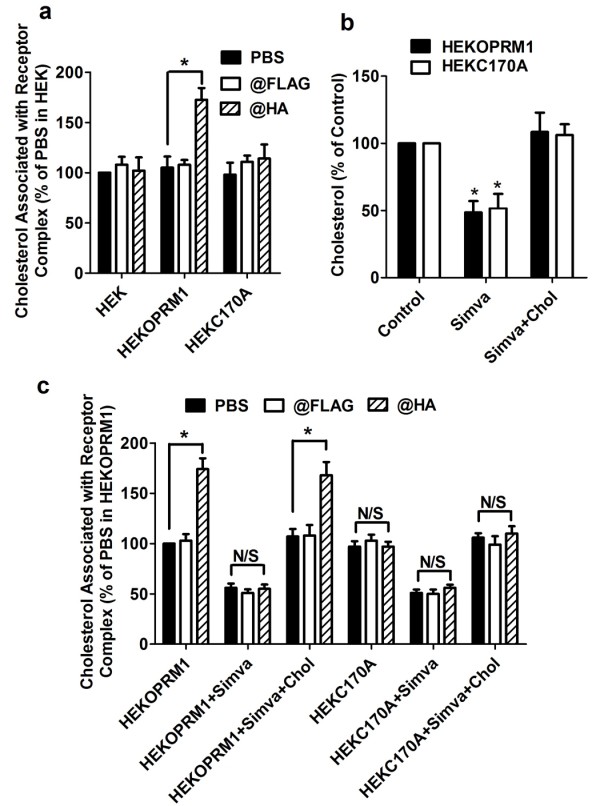
**Palmitoylation facilitates cholesterol association**. (a) Cholesterol associated with receptor complex was determined in HEK, HEKOPRM1 and HEKC170A cells. The amount of cholesterol precipitated with PBS in HEK cells was used for normalization. (b-c) HEKOPRM1 and HEKC170A cells were treated with PBS (Control), 0.5 μM simvastatin (Simva), or 0.5 μM simvastatin with 20 ng/ml cholesterol (Simva+Chol) for 12 h. Membrane cholesterol contents were determined in (b) as described in *Methods*. Cholesterol association with receptor complex was determined in (c). One-way ANOVA with Dunnett's test (b) or two-way ANOVA with Bonferroni's test (a, c) was used. The error bars and "*" represent the standard deviations and significant changes (p < 0.05, n > 3), respectively. N/S, no significance.

Using this procedure, HA antibody precipitated more cholesterol in HEKOPRM1 cells than did FLAG antibody or PBS. Since the receptor was HA-tagged at the N-terminus, any detected cholesterol in the precipitated receptors could be identified as cholesterol associated with receptor signaling complex. Further, in control experiments using HEK cells, the two antibodies and PBS precipitated similar amounts of cholesterol (Figure 5a). These results confirm that this assay detects cholesterol associated with the receptor signaling complex.

We also noted decreased cholesterol association in HEKC170A cells (Figure 5a). The amount of cholesterol precipitated with the HA antibody was similar to that precipitated with the FLAG antibody, suggesting that the mutation on C3.55(170) contributes to the cholesterol association. Although the assay could not distinguish between cholesterol that associates with the receptor directly and cholesterol that associates with other proteins within the signaling complex, receptor palmitoylation appears to regulate the amount of cholesterol that associates with the complex.

### Cholesterol association facilitates homodimerization and Gαi2 coupling

Because 1) a cholesterol-palmitoyl interaction has been suggested in the β2-AR crystal structure, and 2) we demonstrated that receptor palmitoylation facilitates receptor dimerization and G protein coupling of OPRM1 (Figure 2 to Figure 4), we sought to discover whether cholesterol has the same functions in the latter receptor. To determine the contribution of cholesterol association to receptor signaling, we treated the cells with simvastatin, an HMG-CoA reductase inhibitor. We assayed receptor dimerization and G protein coupling with FRET, colocalization and immunoprecipitation.

The cellular cholesterol content decreased on the cell membrane of HEKOPRM1 cells after treatment of the cells with 0.5 μM simvastatin for 12 h. We were able to prevent the decreases in cholesterol content by including 20 ng/ml cholesterol during the simvastatin treatment (Figure 5b). As expected, simvastatin treatment also induced a decrease in cholesterol level on the membrane of HEKC170A cells (Figure 5b).

We also assessed how cholesterol depletion influences its association with the receptor signaling complex. Simvastatin treatment decreased the association of cholesterol with the receptor complex, but this could be prevented by including 20 ng/ml cholesterol in the culture medium (Figure 5c). Further, simvastatin not only decreased the amount of cholesterol precipitated in the "PBS" group, it also impaired the ability of the HA antibody to precipitate more cholesterol than FLAG antibody. Since cholesterol association was not detected in the HEKC170A cells, simvastatin treatment had no effect in these cells (Figure 5c).

**Figure 6:**
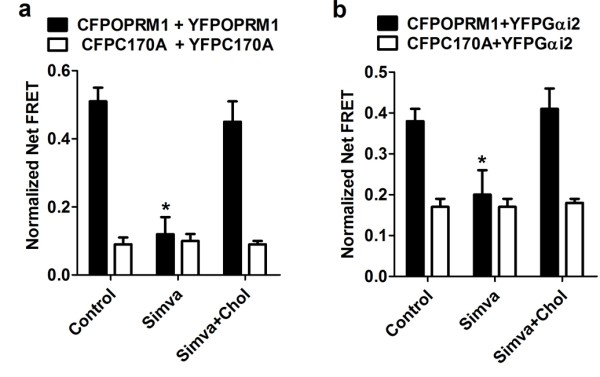
**Reducing cellular cholesterol affects homodimerization and G protein coupling**. (a) HEK cells were transfected with CFPOPRM1 and YFPOPRM1 or transfected with CFPC170A and YFPC170A for 24 h. These cells were than treated with PBS (Control), 0.5 μM simvastatin (Simva), or 0.5 μM simvastatin with 20 ng/ml cholesterol (Simva+Chol) for 12 h. The FRET was analyzed to determine the amount of homodimer. (b) HEK cells were transfected with CFPOPRM1 and YFPGαi2 or transfected with CFPC170A and YFPGαi2 for 24 h. These cells were than treated as in (a) and G protein coupling was determined with FRET. One-way ANOVA with Dunnett's test was used. The error bars and "*" represent the standard deviations and significant changes (p < 0.05, n > 3), respectively.

Since simvastatin treatment decreased the cellular cholesterol content, we used the FRET assay to determine whether cholesterol content affects receptor dimerization and G protein coupling. The normalized net FRET between CFPOPRM1 and YFPOPRM1 in simvastatin-treated HEK cells was decreased compared to untreated cells and could be reversed by inclusion of cholesterol during the simvastatin treatment (Figure 6a). A similar simvastatin-mediated decrease was observed with CFPOPRM1 and YFPGαi2 and could also be reversed by the inclusion of cholesterol during the simvastatin treatment (Figure 6b). However, the cholesterol depletion induced by simvastatin did not affect the homodimerization (Figure 6a) or G protein coupling of C170A (Figure 6b). Therefore, the presence of cholesterol within the receptor signaling complex is critical for receptor homodimerization and Gαi2 coupling.

**Figure 7:**
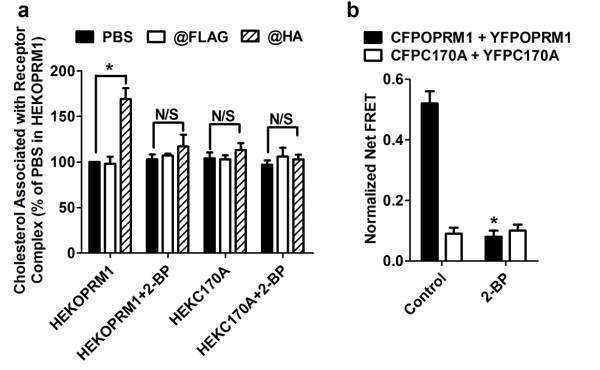
**Palmitoylation inhibitor impairs homodimerization and cholesterol association**. (a) HEKOPRM1 or HEKC170A cells were treated with 50 μM 2-BP or vehicle for 12 h, and the cholesterol associated with receptor complex was measured. (b) HEK cells were transfected with CFPOPRM1 and YFPOPRM1 or transfected with CFPC170A and YFPC170A for 24 h. 50 μM 2-BP or vehicle was used to treat the cells for additional 12 h. FRET was then determined. Two-way ANOVA with Bonferroni's test (a) or student t-test (b) was used. Error bars and "*" represent the standard deviations and significant changes (p < 0.05, n > 3), respectively.

We further illustrated the relationship between receptor palmitoylation, cholesterol association, and receptor dimerization by incubating cells with the palmitoylation inhibitor 2-BP. We observed a decrease in cholesterol associated with the OPRM1 signaling complex after 2-BP treatment (Figure 7a). Because of the inhibitory effect of palmitoylation blockage on Gαi2 membrane targeting [14], the influence of 2-BP on Gαi2 coupling was not investigated. We also saw a reduction in the normalized net FRET between CFPOPRM1 and YFPOPRM1 after 2-BP treatment (Figure 7b). In addition, 2-BP treatment did not affect cholesterol association with C170A or the homodimerization of C170A, since palmitoylation blockage in C170A already impaired these two functions to basal levels (Figure 7).

### Computational modeling suggests that palmitoyl-cholesterol interaction stabilizes the OPRM1 homodimer

We undertook modeling studies to confirm that a specific cholesterol interaction with palmitoylated C3.55(170) may enhance the interactions at the homodimer interface of OPRM1. The OPRM1 model we developed for the modeling studies reported here is a homology model that uses the β_2_-AR crystal structure as a template [15]. As mentioned in *Methods*, OPRM1 has two TMHs that differ in the position of helix deforming residues from the template β_2_-AR (TMH2: P2.58 OPRM1 vs. P2.59 β_2_-AR; TMH4: P4.59 OPRM1 vs. P4.60 β_2_-AR). Our Conformational Memories (CM) calculations revealed that the location of P2.58 in OPRM1 causes the pitch of TMH2 to change after the proline such that residue 2.60 faces into the binding pocket. This same residue position in the β_2_-AR resides in the TMH2/3 interface. These results are consistent with the conformation of TMH2 in the CXCR4 crystal structure (CXCR4 also has a Pro at 2.58) [16]. The TMH4 region from 4.53 to 4.58 is SSAIGLP in OPRM1. Our CM calculations showed that the presence of the G2.56 so close to P2.58 causes a wider turn in TMH4 than is seen in β_2_-AR. The net result is that TMH4 leans more towards TMH5. One result of this change is the lipid exposure of residue 4.59, a key residue in the TMH4 dimer interface (see below). These two key helix changes, along with the resulting changes in helix packing, distinguish the OPRM1 binding pocket (and lipid face) from that of β_2_-AR.

**Figure 8:**
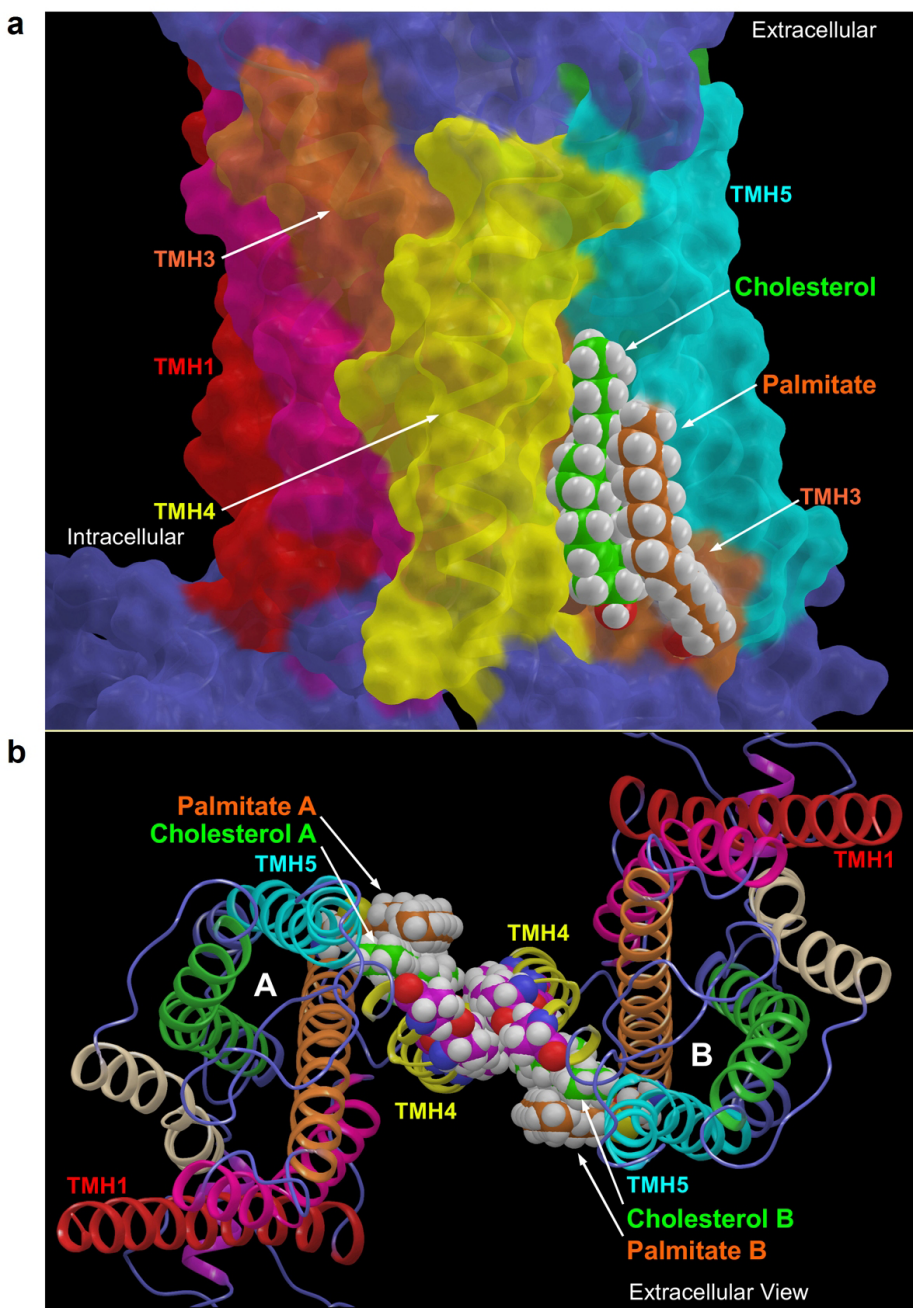
**Computational modeling of the OPRM1 homodimer interface**. (a) This figure illustrates the position of cholesterol relative to the palmitoyl and the OPRM1 TMH bundle. The view is from lipid looking toward TMH4 (yellow). The OPRM1 model is displayed in molecular surface view, with cholesterol (green) and palmitoyl (orange) contoured at their VDW radii. TMH3 and TMH5 are in orange and cyan, respectively. (b) This figure illustrates an extracellular view of protomers A and B forming the OPRM1 homodimer TMH4 interface. Residues that form the interface (N4.41, I4.44, C4.48, I4.51, A4.55, and P4.59) are contoured at their VDW radii and colored magenta. Also contoured at their VDW radii are the palmitoyls (orange) and cholesterols (green). In this arrangement, cholesterols associated with one protomer also interact with the opposite protomer.

Our detailed modeling procedures are described in *Methods*. Figure 8a illustrates the position of cholesterol relative to the palmitoyl and the TMH bundle. Due to the extreme tilt of TMH3 in the TMH bundle, the intracellular end of TMH3 (orange) is between the intracellular ends of TMH4 (yellow) and TMH5 (cyan). This position of TMH3 allows the cholesterol to pack between the C3.55(170) palmitoyl and TMH4. Figure 8b provides an extracellular view of the final energy-minimized OPRM1 homodimer. In the resultant dimer, cholesterol is packed against the TMH4 interface and TMH3. The palmitoyl at C3.55(170) is packed against the cholesterol with TMH5, blocking cholesterol from leaving the interface. Table 3 provides a summary of the resultant interaction energies for the palmitoylated OPRM1 homodimer/cholesterol complex. It is clear here that the major energetic contributions to the interaction energies between the protomers are van der Waals (VDW) energies. The homodimer interface residues with VDW contributions are N4.41, I4.44, C4.48, I4.51, and I4.56, with a total energy of -14.76 kcal/mol. The cholesterol associated with protomer A interacts with protomer B residues R4.40, N4.41, K4.43, I4.44, and V4.47, contributing an additional -2.44 kcal/mol, and the cholesterol associated with protomer B contributes an additional -2.39 kcal/mol. Thus, the total cholesterol interactions (-4.83 kcal/mol) contribute 24.7% to the total interaction energy at the homodimer interface (-19.59 kcal/mol), suggesting that the interaction between cholesterol and palmitoyl facilitates OPRM1 homodimerization

**Table 3:** Homodimer interface interaction energies.

Interaction energies		Coulombic	VDW	Total
		
		kcal/mol	kcal/mol	kcal/mol
**Protomer A, B**				
	T4.38	-0.01	-0.03	-0.04
	P4.39	0.08	-0.02	0.06
	R4.40	0.30	-0.31	-0.01
	**N4.41**	-0.32	-1.06	-1.38
	**I4.44**	-0.02	-3.56	-3.58
	I4.45	0.09	-1.22	-1.13
	V4.47	-0.06	-0.24	-0.31
	**C4.48**	0.11	-1.49	-1.38
	W4.50	0.03	-0.02	0.01
	**I4.51**	0.18	-2.77	-2.58
	L4.52	0.03	-0.11	-0.08
	S4.54	0.03	-0.06	-0.03
	**A4.55**	0.21	-2.11	-1.90
	I4.56	0.11	-2.42	-2.32
	G4.57	-0.03	0.00	-0.04
	**P4.59**	0.01	-0.07	-0.06
**Subtotal**		0.74	-15.50	**-14.76**

**Cholesterol A, Protomer B**		0.07	-2.52	-2.44
**Cholesterol B, Protomer A**		0.10	-2.49	-2.39
**Subtotal**		0.17	-5.01	**-4.83**

**Total**		0.91	-20.51	**-19.59**

Residues in bold are residue positions shown to be involved in the TMH4 homodimer inactive state interface of the dopamine D2 receptor [36].

## Discussion

In summary, our experimental studies suggest that a cholesterol-palmitoyl interaction facilitates homodimerization and G protein coupling. This conclusion is further supported by computational models of the OPRM1 homodimer, which show that palmitoyl linked to C3.55(170) can trap cholesterol at the interface of OPRM1 homodimer; this subsequently stabilizes the homodimerization. C3.55 is highly conserved in the class A rhodopsin GPCRs, especially the peptide, opsin, olfactory, thyrotropin-releasing hormone and melatonin receptor families [17]. C3.55(130) on the melatonin type 1 receptor contributes to G protein activation [18]. The critical role of the homologous residues in G protein coupling/activation has also been confirmed in the m5 muscarinic receptor, the α_1b_-adrenergic receptor, the AT1 angiotensin receptor, and the interleukin-8 receptor, as summarized in the G Protein-Coupled Receptor Data Base (GPCRDB) [17]. Although palmitoylation of the other two opioid receptor, δ-opioid receptor and κ-opioid receptor, has not been reported yet, the palmitoylation site in OPRM1 is conserved in the other two opioid receptors: C3.55(151) in δ-opioid receptor and C3.55(161) in κ-opioid receptor. The location of δ-opioid receptors in cholesterol-rich lipid rafts and its formation of a heterodimer OPRM1 suggests the possible involvement of the palmitoylation on C3.55 in its signaling [19,20].

The palmitoylation inhibitor 2-BP decreased the normalized net FRET between CFPOPRM1 and YFPOPRM1 to the level of that between CFPC170A and YFPC170A (Figure 3a and 7b). 2-BP also decreased the cholesterol association with the signaling complex (Figure 7a). We did not determine the influences of 2-BP on receptor signaling and G protein coupling, because of its inhibitory effect on the membrane targeting of the highly palmitoylated G proteins [14]. Since the effects of 2-BP were consistent with the effects of the C3.55(170) mutation, any conformational changes other than depalmitoylation are not significant in our current observations.

The dimerization of GPCRs, including the higher order oligomeric state of rhodopsin, has been long reported [21,22]. The interface of monomer GPCR has been suggested to be too small for G protein coupling [9]. However, the interaction between GPCR monomer and G protein still cannot be excluded. Here, the FRET between CFPC170A and YFPC170A was about 13% of that between CFPOPRM1 and YFPOPRM1 (Figure 3a), whereas the FRET between CFPC170A and YFPGαi2 was about 28% of that between CFPOPRM1 and YFPGαi2 (Figure 4a). This difference suggests that the receptor monomer can still interact with Gαi2, though with a lower affinity than the homodimer.

In the β_2_-AR crystal structure, cholesterol is found situated at the intracellular side of the TMH1-TMH4 bundles [1]. Thus, it is probable that localization of OPRM1 within cholesterol-enriched domains such as lipid rafts regulates the cholesterol content within the receptor complex and receptor signaling. Therefore, the cholesterol-enriched lipid raft microdomain may be essential for the ability of the cholesterol-palmitoyl interaction to stabilize receptor homodimerization and G protein coupling. In our studies, C3.55(170) palmitoylation affected the amount of cholesterol associated with the signaling complex. As reported previously, Gαi2 anchors OPRM1 to the lipid raft microdomains. Thus, by facilitating Gαi2 coupling, cholesterol associated with OPRM1 increases the percentage of receptor in lipid raft microdomains.

The modeling studies reported here show that the C3.55(170) palmitoylation site is located very near the Class A GPCR inactive homodimer interface identified by Guo and colleagues for dopamine D2 receptor [23]. For an OPRM1 homodimer formed at this interface, the cholesterol associated with C3.55(170) is ideally placed to contribute to the total energy of interaction for the homodimer. As Lambert discusses in his recent review [24], the interaction energies at the homodimer interface are likely weak but sufficient to promote dimer formation transiently. We report here that the enthalpic component of the interaction between OPRM1 homodimers is -14.76 kcal/mol and that the presence of cholesterol at the OPRM1 homodimer interface raises the total interaction enthalpy to -19.59 kcal/mol. This modest interaction energy is derived predominantly from VDW interactions, as would be expected for the hydrophobic residues as well as the hydrophobic cholesterols in the homodimer interface. By identifying a consensus cholesterol binding motif in the TMH2-TMH4 region that predicts cholesterol binding for 44% of human class A receptors, Hanson and co-workers suggest that specific sterol binding is important to the structure and stability of many GPCRs [25]. However, this consensus motif is not present in OPRM1 and does not involve C3.55.

In conclusion, both our experimental data and computational models delineate a cascade from cholesterol-palmitoyl interaction to receptor homodimerization and then to G protein coupling/activation. Conceivably, by regulating the cholesterol-palmitoyl interaction, either by the control of cholesterol metabolism or receptor palmitoylation, the stability of GPCR homodimers is altered, leading to the uncoupling of G protein. In this respect, the cellular cholesterol content, specifically the cholesterol associated with the receptor, represents an additional target through which the signaling of GPCRs can be regulated.

## Conclusions

### C3.55(170) is the palmitoylation site of OPRM1

OPRM1 is highly palmitoylated. The C3.55(170) has been indirectly suggested as the palmitoylation site, as it has been established that the only other two cysteines [C7.63(346) and C7.68(351)] are not the palmitoylation site [3]. Our current studies provide the first direct evidence that C3.55(170) is the palmitoylation site of OPRM1.

### A cholesterol-palmitoyl interaction can be identified in OPRM1 complex

A cholesterol-palmitoyl interaction has been identified both in the β_2_-AR crystal structure [1] and, now, in OPRM1. Although this is not the first identification of a cholesterol-palmitoyl interaction in a GPCR, our studies suggest that such interactions may be observed in the signaling complexes of many GPCRs.

### Cholesterol-palmitoyl interactions contribute to OPRM1 signaling by facilitating receptor homodimerization and G protein coupling

Our studies also represent the first report on the contributions of cholesterol-palmitoyl interaction to receptor signaling. In addition, by the using multiple assays, including FRET, we illustrate the mechanism underlying these contributions. This understanding provides additional information on receptor homodimerization and G protein coupling.

### Computational modeling of OPRM1 homodimer supports the conclusions listed above

To support the conclusions mentioned above, we generated a computational model of OPRM1 homodimer based on the structure of other relevant GPCRs. Our model suggests that the cholesterol-palmitoyl interaction provides additional energy to stabilize the homodimer, which is consistent with our other observations.

## Methods

### Palmitoylation assay

The palmitoylation assay was carried out as reported by Drisdel et al. [13]. Briefly, receptor was immunoprecipitated with protein G agarose beads. The beads were then sequentially incubated with 50 mM NEM to block free sulfhydryl groups, 1 M hydroxylamine to remove thioester-linked palmitic acid, and 40 μM btn-BMCC to conjugate biotin to the depalmitoylated cysteines. To assess the receptor palmitoylation level, the amount of conjugated biotin was determined by immunoprecipitation and immunoblotting [12,26]. Protein concentrations and receptor expression levels were measured to ensure that equal amounts of receptor were loaded in each lane of the gel.

### Membrane purification and cholesterol assay

Cells were homogenized in 0.32 M sucrose and 10 mM HEPES (pH 7.7). The crude lysate was then centrifuged at 1,000 × *g *for 10 min at 4°C, the supernatant was collected, and the pellet was re-homogenized. These processes were repeated until the pellet appeared translucent. The collected supernatant was centrifuged at 100,000 × *g *for 60 min at 4°C. The pellet was re-suspended and used to determine the cholesterol content in cell membranes. The results were normalized against cholesterol levels in cells under control condition. Cholesterol concentrations were determined by using the Amplex Red Cholesterol Assay Kit (Invitrogen, Carlsbad, CA) on the cell membrane preparation according to manufacturer's instructions.

To determine the amount of cholesterol associated with the OPRM1 complex, a new method was used. Cells were first treated with lysis buffer (50 mM Tris-HCl, pH 7.5, 150 mM NaCl, 0.25% sodium deoxycholate, 0.1% Nonidet P-40, 0.5% Triton X-100, 0.1% digitonin, 50 mM NaF, 1 mM dithiothreitol, 0.5 mM phenylmethylsulfonyl fluoride, 50 mM sodium pyrophosphate, 10 mM sodium vanadate, and 1X protease inhibitor cocktail; Roche, Indianapolis, IN). The supernatants from the cell lysates were divided into three equal aliquots. These aliquots were used to perform co-immunoprecipitation with PBS (control), HA antibody (Convance, 1:1000) (to detect HA-tagged OPRM1 and C170A), or FLAG antibody (Sigma, 1:1000). After antibody incubation, protein G agarose (Invitrogen, Carlsbad, CA) was added for an additional overnight incubation. The resulting agarose was used to determine the amount of precipitated cholesterol with the Amplex Red Cholesterol Assay Kit (Invitrogen, Carlsbad, CA). The FLAG antibody was used as a control antibody to exclude any possible influence of antibody usage. The greater amount of cholesterol precipitated by HA antibody compared with PBS or FLAG antibody reflects the cholesterol associated specifically with OPRM1 signaling complex. Although this method does not directly determine cholesterol's interaction with the receptor, it does specifically detect cholesterol's interaction with the receptor signaling complex.

### FRET

CFP and YFP were fused to the C terminus of wild-type OPRM1 or the C170A mutant of OPRM1. YFPGαi2 has YFP inserted between residues 91 and 92 of Gαi2 [27]. Throughout the studies, all FRET values are expressed as the normalized net FRET using the following formula: I_FRET _= [(I_CFP _× CoA) - (I_YFP _× CoB)]/[the square root of (I_CFP _× I_YFP_)]. I_FRET _is the fluorescence intensity when a CFP-YFP (excitation-emission) filter set is used, I_CFP _is the fluorescence intensity when a CFP-CFP filter set is used, and I_YFP _is the fluorescence intensity when a YFP-YFP filter set is used. CoA was determined in the cells transfected with only CFP constructs by the following formula: CoA = I_FRET _/I_CFP_. CoB was determined similarly. Including "square root" in the formula eliminates the influence from the differential expression of CFP- and YFP-conjugated protein. Briefly, more than twenty individual regions on the cell membrane of a single cell were analyzed, and more than twelve individual cells were analyzed for each sample.

### OPRM1 binding assay

The amounts of receptor on the cell membrane and the affinity of agonists for receptors were determined by binding assay [28]. Briefly, purified cell membrane was incubated with [^3^H]-diprenorphine and agonists/antagonists. After incubation, PEG8000 and NaCl were added to trap the receptors on Whatman GF/B filters for final radioactive reading. Scatchard analyses were carried out to determine the level of wild-type or mutant OPRM1 expressed on cell membranes. To determine the affinities of various ligands, the cell membranes were incubated with 2 nM [^3^H]-diprenorphine and various concentrations of morphine, naloxone, and CTOP (0.01 nM - 10 μM). These competition binding studies were analyzed with one- or two-site curve-fitting models in GraphPad 5.0.

### Transient transfection

The pCMV-shuttle vector (Stratagene) was used in all studies. cDNA generation from the receptors, Gαi2, and their fluorescence-conjugated constructs was controlled by the CMV promoter. Transient transfections were performed with Lipofectamine 2000 (Invitrogen) following manufacturer's instructions. Cells were allowed to rest for 24 h before further treatment.

### Assays based on antibodies

Immunoblotting and co-immunoprecipitation assays were performed as described previously [26]. The same Confocal Imager used for FRET was used to analyze colocalization. Adenylyl cyclase inhibition was measured as previously reported [12]. ERK phosphorylation was determined by immunoblotting [26].

Colocalization studies were performed as reported previously [12]. Briefly, cells were cultured on poly-lysine-coated coverslip in six-well plates. After transient transfection and various treatments, cells were fixed with 2% formaldehyde for 30 min. HA, Flag, and Gαi2 antibodies were used as primary antibodies (1:1000). The confocal images were captured with a BD CARV II Confocal Imager and a Leica DMIRE2 fluorescence microscope. Colocalization of the fluorescence pixels was calculated with IPlab 4.0 software (BD Biosciences-Bioimage) and the following formula: 2 × N_yellow _/(N_red _+ N_green_), where *N *represents the number of pixels with fluorescence intensity over a pre-defined threshold.

### Development of the OPRM1 homodimer model

A computational model of the OPRM1 inactive state was developed using the β_2_-AR crystal structure as a template [1] with two major modifications. First, the TMH 7/elbow/Hx8 region of the β_2_-AR was replaced with that of the adenosine A_2A _crystal structure [29] because the "elbow region" between TMH7 and the C terminus Helix 8 contains only two residues (P7.57 and D7.58). This results in an elbow region of β_2_-AR that is stretched. We would not expect OPRM1 to have a similar conformation since it has three elbow residues (D7.57 E7.58 N7.59). We therefore used the elbow conformation in the A_2A _crystal structure, which also has three residues [29], in the OPRM1 model. Second, the Monte Carlo/simulated annealing technique CM [30] was used to study the conformations of three OPRM1 TMHs with important sequence divergences from the β_2_-AR template: TMH2 (P2.58 OPRM1 vs. P2.59 β_2_-AR), TMH4 (P4.59 OPRM1 vs. P4.60 β_2_-AR), and TMH6 (CWTP OPRM1 vs. CWLP β_2_-AR). The CM technique explores the low free energy conformations possible for a helix of interest using Monte Carlo simulated annealing. The method of CM, developed by Guarnieri and Wilson [31] and extended by Guarnieri and Weinstein [32], efficiently and completely explores the dihedral conformational space of a molecule, independent of the dihedral conformation of the initial molecular structure. The CM method combines Monte Carlo exploration of the dihedral angle space with simulated annealing (MC/SA) to determine the range of values that each dihedral angle is capable of reaching in a broad temperature range. The CM method has been expanded to allow variation of bond angles in addition to dihedral angles [30].

In the CM calculations reported here, the backbone dihedrals of each helix were set to the standard φ (-63°) and ψ (-41.6°) for transmembrane helices. Our established protocol is to allow all torsion angles to vary ± 10°, and to allow a larger variation of ± 50° in regions containing flexible areas. These flexible areas are regions where there are known helix bending residues such as prolines, glycines, serines and/or threonines [33]. The OPRM1 TMH regions considered flexible were the following: TMH2: region of i (P2.58) to i-4 (T2.54); TMH4 region of i (P4.59) to i-4 (A4.55) and TMH6 region of i (P6.50) to i-4 (V6.46). Individual bond angles were allowed to vary ± 8°.

CM calculations are performed in two phases: an exploratory phase and a biased annealing phase. In the exploratory phase, a random walk is used to first identify the region of conformational space most probable for each torsion angle and bond angle. Specifically, each step consists of varying two dihedral angles and one bond angle chosen at random from the entire set of variable angles. The torsion angles and bond angles are randomly picked at each temperature and each move is accepted or rejected using the Metropolis criterion [34]. Accepted conformations in the exploratory phase are used to create "memories" of torsion angles and bond angles that were accepted. This information provides a map of the accessible conformational space of each TMH as a function of temperature. In the biased annealing phase, the only torsion angle and bond angle moves attempted are those that would keep the angle in the "populated conformational space" mapped at 310 K in the exploratory phase.

Here, the initial temperature for each run was 3000 K with 50,000 Monte Carlo steps applied to each torsion or bond angle variation, with cooling in 18 steps to a final temperature of 310 K. The biased annealing phase for the calculations began at 749.4 K, and the cooling to 310 K was performed in 7 steps. 105 structures were output at 310 K. The output from each TMH study was superimposed on the corresponding template helix in the β2-AR template that had been mutated to the sequence of OPRM1. A helix was selected for inclusion in the revised OPRM1 that fit in the bundle with no VDW overlaps with residues on other TMHs.

The CM helices chosen for substitution into the TMH bundle had the following helix bend angles, wobble angles, and face shifts: TMH2 (35.2°, -105.8°, 40.3°), TMH4 (14.8°, -126.1°, 25.9°), and TMH6 (30.6°, -129.9°, 45.6°). Extracellular and intracellular loops were added using MODELLER v8.2 [35]. Energy minimizations were performed using Macromodel and the OPLS2005 all-atom force field (version 9.8, Schrödinger, LLC, New York, NY). A distance-dependent dielectric, 8.0 Å extended nonbonded cutoff, 20.0 Å electrostatic cutoff, and 4.0 Å hydrogen bond cutoff were used. A palmitoyl was added to C3.55(170) and a cholesterol was docked between palmitoylated C3.55(170) and TMH3. Interactive docking studies in Maestro (version 9.1, Schrödinger, LLC, New York, NY) were used to orient two OPRM1/cholesterol protomers at the symmetric TMH4 interface of mouse dark state rhodopsin [22]. In this orientation, the OPRM1 protomers form an interface analogous to that shown by Guo and co-workers to characterize the inactive state homodimer interface of the dopamine D2 receptor [36]. This interface in OPRM1 involves N4.41, I4.44, C4.48, I4.51, A4.55, and P4.59 on each protomer. In the resultant dimer, cholesterol is packed against the TMH4 interface and TMH3. The palmitoyl at C3.55(170) is packed against the cholesterol and TMH5, blocking cholesterol from leaving the interface.

The energy of the OPRM1 homodimer complex was minimized using the same force field, dielectric, and cutoffs as described above. In the first stage of the calculation, Polak-Ribier conjugate gradient minimization was employed until a gradient of 0.1 kcal/mol A^2 ^was reached. A force constant of 250 kcal/mol was used on the loop backbone atoms. All charged residues in the loop regions and at the ends of the TMHs that face toward lipid headgroups were mutated to neutral forms. Non-moving fixed atom restraints were applied to the C-alpha atoms of TMH3 in both protomers, restraining the protomers from moving apart. The protocol was repeated with TMH3 non-moving fixed restraints removed.

Macromodel was used to output the pair-wise interaction energy (VDW and coulombic) for a given pair of atoms. The nonbonded interactions are represented in OPLS2005 (as implemented in Macromodel through Coulomb and Lennard-Jones terms) interacting between sites centered on nuclei. Thus, the intermolecular interaction energy between molecules *a *and *b *is given by the sum of interactions between the sites on the two molecules [37], as represented in the following equation:

$$\Delta {\text{E}}_{ab}=\overset{on\;a}{\underset{i}{\sum }}\overset{\;on\;b}{\underset{j}{\sum }}\left(q_{i}q_{j}e^{2}/)r_{ij}+A_{ij}/)r_{ij}^{12}-C_{ij}/)r_{ij}^{6}\right)$$

where *a *and *b *are collected by Macromodel as atom sets representing all atoms of a single residue for *a *and all atoms of a nearby residue for *b*. The residue represented by *a *is evaluated separately against all residues within a 7.0Å radius of residue *a*. With cholesterol A defined as group 1, and with all of the atoms of any residue within 7.0 Å of protomers A or B defined as group 2, the pair-wise interaction energies were calculated. The interaction energy at the homodimer interface was calculated as the sum of the interaction energies between protomers A and B at the homodimer interface plus the interaction energy of cholesterol A with protomer B and the interaction energy of cholesterol B with protomer A. Cholesterols A and B were blocked from interacting with each other by the close interactions and steric bulk of protomer A's TMH4 and protomer B's TMH4; the palmitoyls could not interact with each other for the same reason.

## Statistics

Experiments were repeated at least four times (with more than twelve individual cells used for image analysis). Data were analyzed and compared by one-way ANOVA with Dunnett's test as a post-hoc test for comparisons, or by two-way ANOVA with the Bonferroni's test as a post-hoc test. Error bars and "*" represent the standard deviations and significant changes (p < 0.05), respectively.

## List of abbreviations

2-BP: 2-bromopalmitate; β2-AR: β2-adrenergic receptor; btn-BMCC: 1-biotinamido-4- [4'ss-(maleimidomethyl) cyclohexane carboxamido] butane; CM: Conformational Memories; CTOP: Cys2-Tyr3-Orn5-Pen7-amide; FRET: fluorescence resonance energy transfer; GM1: monosialotetrahexosylganglioside; GPCR: G protein-coupled receptor; NEM: N-ethylmaleimide; OPRM1: μ-opioid receptor; TMH: transmembrane helix; TR: transferrin receptor; VDW: van der Waals.

## Competing interests

The authors declare that they have no competing interests.

## Authors' contributions

HZ, PHR, and PYL participated in research design.

HZ, EBP, DPH, YuZ, JiC, and YaZ conducted experiments.

HZ, EBP, and DPH performed data analysis.

HZ, EBP, PHR, HHL, and PYL wrote or contributed to the writing of the manuscript.
